# Surface faceting and elemental diffusion behaviour at atomic scale for alloy nanoparticles during *in situ* annealing

**DOI:** 10.1038/ncomms9925

**Published:** 2015-11-18

**Authors:** Miaofang Chi, Chao Wang, Yinkai Lei, Guofeng Wang, Dongguo Li, Karren L. More, Andrew Lupini, Lawrence F. Allard, Nenad M. Markovic, Vojislav R. Stamenkovic

**Affiliations:** 1 Center for Nanophase Materials Sciences, Oak Ridge National Laboratory, One Bethel Valley Road, Building 4515, Oak Ridge, Tennessee 37831-6064, USA; 2 Department of Chemical and Biomolecular Engineering, Johns Hopkins University, Baltimore, Maryland 21218, USA; 3 Department of Mechanical Engineering and Materials Science, University of Pittsburgh, Pennsylvania 15260, USA; 4 Materials Science Division, Argonne National Laboratory, Argonne, Illinois 60439, USA; 5 Materials Science and Technology Division, Oak Ridge National Laboratory, Oak Ridge, Tennessee 37831, USA

## Abstract

The catalytic performance of nanoparticles is primarily determined by the precise nature of the surface and near-surface atomic configurations, which can be tailored by post-synthesis annealing effectively and straightforwardly. Understanding the complete dynamic response of surface structure and chemistry to thermal treatments at the atomic scale is imperative for the rational design of catalyst nanoparticles. Here, by tracking the same individual Pt_3_Co nanoparticles during *in situ* annealing in a scanning transmission electron microscope, we directly discern five distinct stages of surface elemental rearrangements in Pt_3_Co nanoparticles at the atomic scale: initial random (alloy) elemental distribution; surface platinum-skin-layer formation; nucleation of structurally ordered domains; ordered framework development and, finally, initiation of amorphization. Furthermore, a comprehensive interplay among phase evolution, surface faceting and elemental inter-diffusion is revealed, and supported by atomistic simulations. This work may pave the way towards designing catalysts through post-synthesis annealing for optimized catalytic performance.

## Abstract (web summary)

Post-synthesis annealing can tailor surface configurations of nanoparticles. Here, the authors use *in situ* aberration-corrected scanning transmission electron microscopy to track the evolution of individual alloyed metal nanoparticles at atomic scale and discern five distinct stages of surface rearrangement.

## Graphical abstract

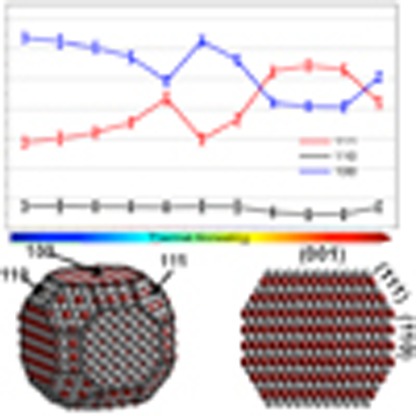

Pt-bimetallic (PtM where M=Fe, Co, Ni and so on) nanoparticles (NPs) have attracted great interest because of their outstanding activity for the oxygen reduction reaction (ORR) in fuel cell applications^1^,^2^. Such PtM NPs exhibit enhanced catalytic performance compared with commercial Pt/C fuel cell catalysts, with improvement factors varying from 2 × to greater than 20 × depending on the particle size, morphology, composition and surface structure. Each of these material parameters can influence and/or control the geometric and electronic structure of the particle surface, and therefore play a vital role in determining the catalytic performance of the PtM catalysts^3^,^4^,^5^,^6^,^7^,^8^,^9^.

Post-annealing thermal treatments have long been employed as an effective strategy to tailor the material parameters and optimize catalytic behaviour of PtM NPs, especially for controlling the NP size, surface faceting and near-surface elemental distributions. Enhanced catalytic performance has been reported for annealed PtM NPs, which could be directly correlated with the specific surface atomic configuration created during thermal annealing^5^,^10^,^11^,^12^. For example, it was reported that the ORR activity of monodisperse Pt_3_Co NPs was enhanced by a factor of 3–4 × versus Pt/C after mildly annealing at ∼500 °C, which was attributed to the formation of a Pt-enriched surface layer and Co-enriched subsurface, with the resultant activity increase due to modification of the surface electronic structure^11^. In another study, it was reported that thermal annealing of a commercial Pt_3_Co/C catalyst at 700 °C generated intermetallic ordering of the Pt_3_Co NP core and the formation of a pure 0.5-nm-thick Pt shell, significantly improving both the ORR activity and durability compared with the initial disordered alloy structure^12^. The atomic structures of these catalysts, as well as other NP catalysts, were typically characterized by imaging the post-annealed NP structures or by imaging different NPs after specific annealing temperatures to elucidate the mechanism(s) responsible for the observed improvement in catalytic performance. As a result, the intermediate atomic-level structural and compositional NP transitions, as well as the underlying mechanisms responsible for these changes, remain elusive. Most importantly, the non-systematic, trial-and-error approaches used thus far to explore thermal annealing effects have missed unique, metastable atomic configurations that could introduce novel physical or chemical properties. Greater understanding can be gained from time-resolved atomic-resolution *in situ* studies^13^ to track elemental diffusion and atomic structural evolution in the same, individual NPs during the annealing process.

Here, we report on the structural and compositional transitions observed for Pt_3_Co NPs during *in situ* scanning transmission electron microscopy (STEM) annealing experiments. These studies are conducted by tracking the dynamic evolution of NP morphology, faceting, elemental segregation, phase transition and strain distribution for individual NPs during annealing as a function of temperature and time, using high-angle annular dark-field (HAADF) STEM imaging. This study conceptualizes the use of simple annealing treatments to manipulate the atomic configuration of alloy NPs, thereby facilitating the tailoring of their physical and chemical properties for catalytic applications.

## Results

### *In situ* annealing and imaging in a STEM

**Figure 1:**
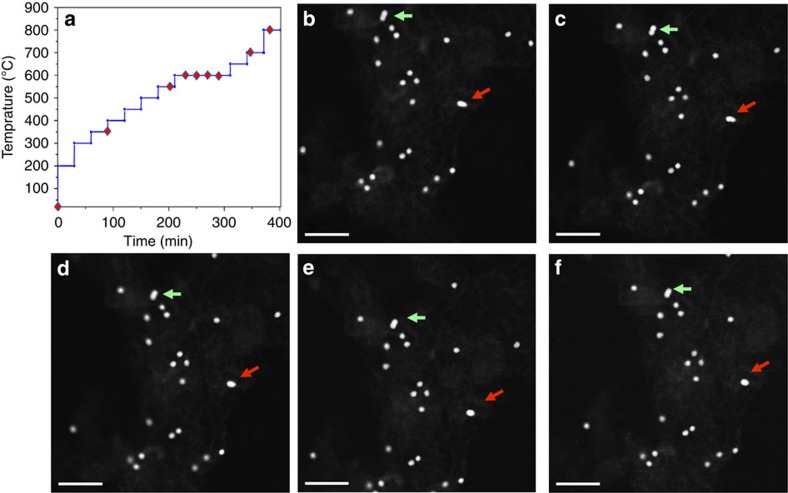
*In situ* annealing sequence. (**a**) The annealing sequence for *in situ* experiments, plotted as the thermal annealing temperature with respect to time. The specific annealing conditions for the captured HAADF-STEM images shown in Fig. 2 are labelled with red diamonds on this plot. (**b**–**f**) Low-magnification HAADF-STEM images of Pt_3_Co NPs supported on carbon black annealed at different temperatures. No obvious NP coalescence was observed until a temperature of 600 °C was reached, even for overlapping particles (shown by arrows) at RT. Scale bar, 50 nm.

The Pt_3_Co NPs in this study were supported on a commercial carbon black support^14^. The Pt_3_Co/C were dispersed on a holey carbon film supported across a Protochips Aduro heating device (details provided in the Supplementary Information section). The *in situ* annealing experiments were performed in an aberration-corrected STEM (JEOL 2200FS). The sequence of annealing steps is shown in Fig. 1a, where the temperature was raised to 800 °C over 400 min. Low-magnification HAADF-STEM images acquired at different temperatures (Fig. 1b–e) showed no obvious NP coalescence until a temperature of 600 °C was reached, which is a higher temperature than Pt NP coalescence temperatures (400–500 °C) reported in the literature^11^. This higher coalescence temperature is attributed to the NP size and compositional homogeneity, as well as the high monodispersion of NPs on the carbon black support. In fact, NPs in direct contact with each other (marked with arrows in Fig. 1b–f) did not exhibit evidence for coalescence until a temperature of 600 °C was reached (Fig. 1e). Additional images showing the homogeneity and monodispersity of the pristine Pt_3_Co NPs are presented in Supplementary Fig. 1.

**Figure 2:**
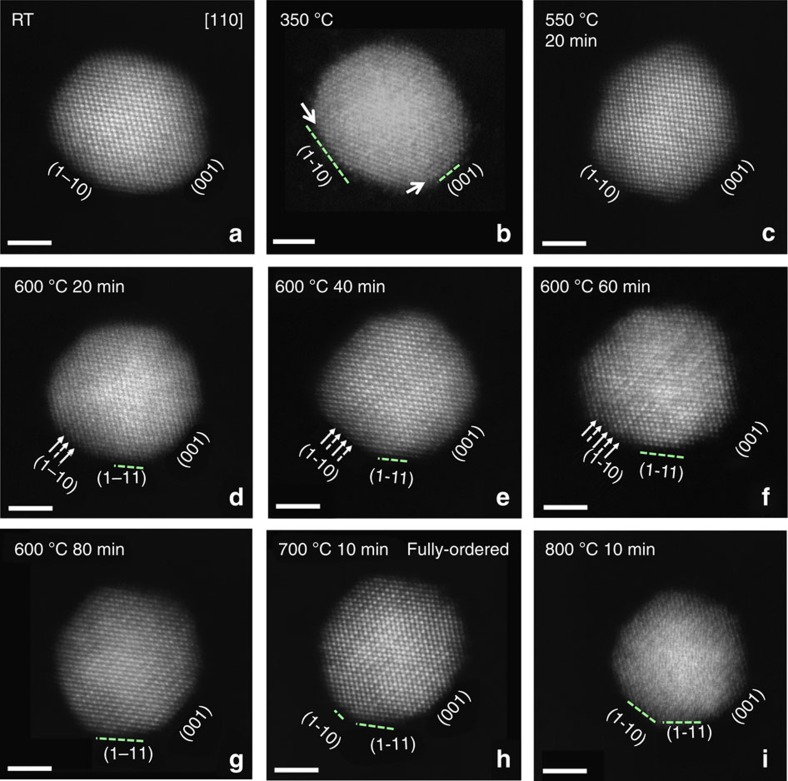
Atomic-resolution HAADF-STEM images. (**a**–**i**) Atomic-resolution HAADF-STEM images of a single Pt_3_Co NP acquired at different temperature and annealing times. The (1–10), (111) and (001) surface facets are labelled for each annealing stage. The alternating planes of bright-dark contrast corresponding to the formation of the ordered Pt_3_Co structure, are marked by arrows in **d**, indicating the origin of transformation of fcc to L1_2_ phase. Scale bar, 2 nm.

Multiple representative Pt_3_Co NPs were selected to track the atomic-scale structural and compositional changes occurring during isothermal holds for times ranging from 10 to 80 min at several different temperatures (room temperature (RT) to 800 °C) during the *in situ* annealing sequence (Fig. 1a). A series of HAADF-STEM images of a single Pt_3_Co particle captured during *in situ* heating are shown in Fig. 2 (with the simultaneously acquired BF-STEM images shown in Supplementary Fig. 2). An initial composition of 3:1 for the Pt:Co NP shown in Fig. 2a was confirmed using energy dispersive X-ray spectroscopy (EDS; see Supplementary Fig. 3). As there is a relatively large difference in the atomic number of Pt and Co, the contrast present in the atomic-scale HAADF-STEM images can be used to estimate the elemental concentrations of Pt and Co comprising each atomic column projection in the image^15^,^16^.

### Surface platinum segregation

**Figure 3:**
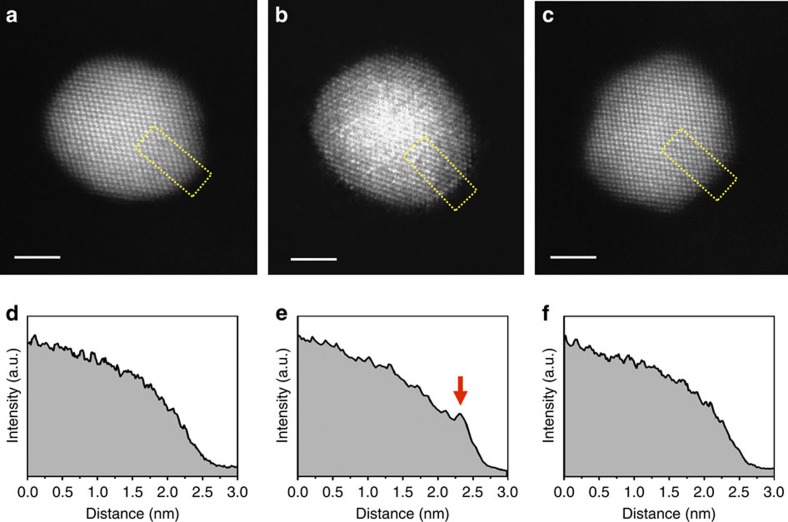
Pt surface segregation. (**a**–**c**) HAADF-STEM images of the Pt_3_Co NP at different annealing temperatures, (**a**) RT, (**b**) 350 °C and (**c**) 550 °C, used for elemental segregation assessments; (**d**–**f**) corresponding intensity profiles of **a**–**c** across particle images, which reveals Pt surface segregation behaviour. The dashed boxes shown in images indicate locations where the intensity profiles were obtained. An obvious intensity increase at the NP surface in **e** marked with an arrow indicates surface segregation of Pt at 350 °C, which is absent at RT and 550 °C.

The HAADF-STEM image of the Pt_3_Co NP at RT (Fig. 2a) exhibits brighter contrast at the centre of the NP, which gradually changes to darker contrast towards the NP surface; this image contrast variation follows the thickness change across the spherical NP. The contrast uniformity within areas of the same NP thickness is consistent with a random distribution of Pt and Co atoms in the Pt_3_Co alloy NP. No distinct faceting was observed on the initial NP surface at RT (Fig. 2a). Increasing the temperature to 350 °C resulted in Pt segregation to the surface, as evidenced by the appearance of a slightly brighter contrast on the NP surface compared with that at RT (shown by arrows in Fig. 2b). Pt surface segregation is clearly demonstrated from image intensity profiles taken across the HAADF-STEM image of the NP (Fig. 3a–f), exploiting the sensitivity of HAADF-STEM imaging with respect to the atomic number^5^,^16^. As shown in the corresponding HAADF-STEM image and intensity profile, Fig. 3b,e, respectively, an increase in the image contrast was found directly at the surface of the NP annealed at 350°C compared with that at RT (Fig. 3a,d, respectively), supporting segregation of Pt atoms to the surface.

**Figure 4:**
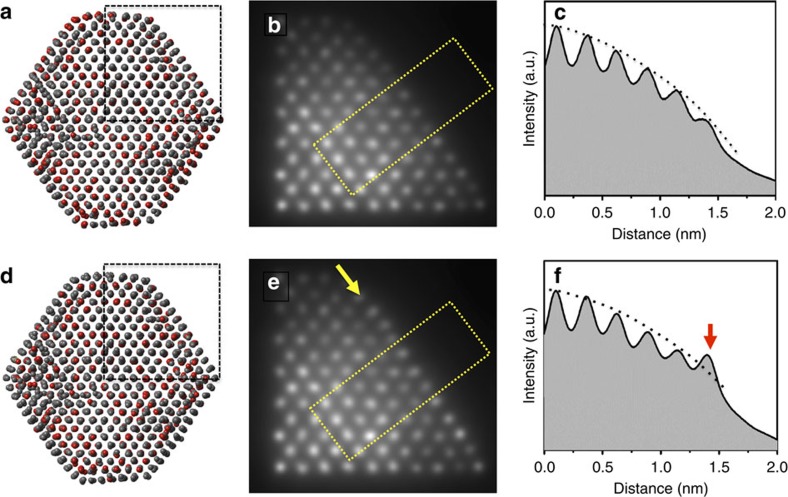
Multi-slice simulations. Multi-slice simulations of HAADF-STEM images of relaxed alloyed Pt_3_Co NPs performed for a particle size of 5.3 nm. (**a**,**d**) Calculated structure models without and with Pt surface segregation. Corresponding simulated images (**b**,**e**) and intensity profiles across simulated images (**c**,**f**). Dashed boxes shown in **b** and **e** indicate the locations where the intensity profiles were measured. Arrows in **e** and **f** mark the contrast increase, which results from Pt surface segregation.

Multi-slice simulations further proves that such a contrast change at the NP surface is a result of Pt segregation. Structural models of a Pt_3_Co NP, with and without Pt segregation, were calculated, and the corresponding HAADF-STEM images were simulated using multi-slice methods (Fig. 4). A particle size of 5.3 nm was used in the calculations to reduce the computing time. The structural models were based on a randomly alloyed Pt_3_Co with structural relaxations. The Pt_3_Co NP structure with Pt segregation (Fig. 4d) was obtained by energy-biased Metropolis Monte Carlo (MC) simulations at 350 °C. Detailed descriptions regarding the calculation and simulation methods are described in the Supplementary Information. The simulated HAADF-STEM images (Fig. 4b,e) show relatively clear atomic columns compared with the experimental images shown in Fig. 2 because of the smaller particle size used for the simulations. Nevertheless, an obvious difference in contrast was revealed in the intensity profiles from the surface atomic columns between the NP without and with Pt segregation, as shown in Fig. 4c,f, respectively, which are consistent with the NP behaviour observed experimentally (Fig. 3).

**Figure 5:**
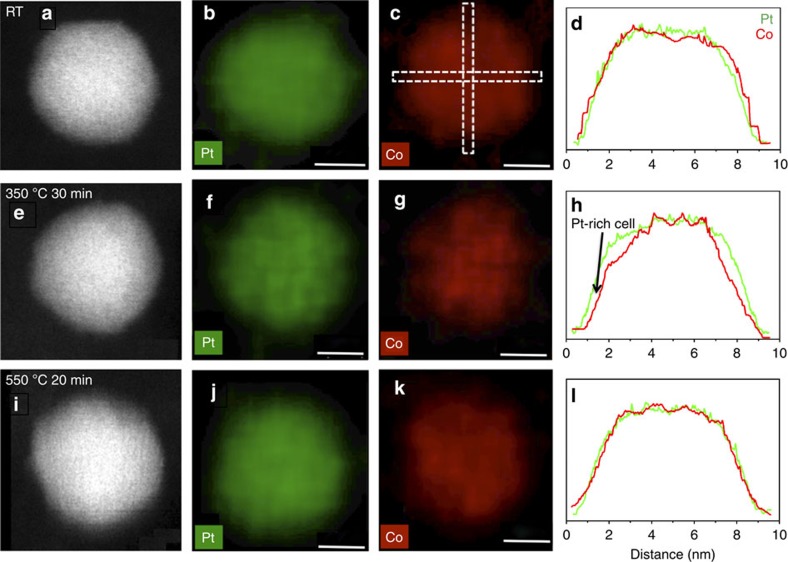
EDS elemental mapping. HAADF-STEM images and corresponding EDS elemental maps of a Pt_3_Co nanoparticle, which was annealed at different temperatures: RT (**a**–**d**), 350 °C (**e**–**h**) and 550 °C (**i**–**l**). The intensity profiles of Pt and Co (rightmost column) are aligned to the maximum of Pt and Co intensities in each sub-figure and demonstrate that Pt segregates to the particle surface at 350 °C and diminishes when NP is annealed at 550 °C. These intensity profiles are the average of intensity profiles at perpendicular and horizontal directions (schematically shown as dashed boxes in the Co map at RT). Scale bar, 4 nm.

The Pt-enriched surfaces disappear when the annealing temperature is increased from 350 to 550 °C (Figs 2c and 3c,f), behaviour that was also observed for other Pt_3_Co NPs (Supplementary Fig. 4) in the same sample. These results show that Pt-segregation to the NP surface is extremely sensitive to the annealing temperature and is explained by the interplay between surface segregation and the compositional disorder present in the as-synthesized Pt_3_Co NPs. The segregation of Pt to the NP surface is mainly driven by the decrease in free energy owing to a negative surface segregation enthalpy^17^, which will lead to a decrease in configurational entropy of the system by inducing surface compositional ordering. Although surface-segregation free energy eventually turns positive because of the increased entropic contribution, increasing the annealing temperature results in the observed disappearance of the Pt-segregated surface. The surface segregation behaviour of Pt was confirmed by EDS mapping of the NPs at 350 and 550 °C, as shown in Fig. 5a–d for a different annealed Pt_3_Co NP from the same sample. The Pt_3_Co NP exhibits a homogenous random elemental distribution at RT; Pt surface enrichment is observed when the NP is heated to 350 °C (Fig. 5e–h), but vanishes when the temperature is further increased to 550 °C (Fig. 5i–l). Careful selection of the NP annealing temperature is critical to form a Pt-segregated surface architecture for enhanced catalytic activity.

Surface faceting accompanies the elemental diffusion observed for the NPs. Distinct {110} surface facets form at 350 °C (shown by arrows in Fig. 2b), the development of which are coincident with the observed Pt surface segregation. Surface faceting occurs as a combined result of Pt segregation to the surface and the initiation of atomic surface reconstruction during the early stage of annealing, which is consistent with previous theoretical calculations^11^. Well-defined facets are observed at 550 °C compared with those formed at lower temperatures (350 °C), which is likely a result of enhanced elemental diffusion at the elevated temperature. Interestingly, {001} faceting was more prominent, whereas {110} facets shrink, when the temperature is increased from 350 to 550 °C, indicating a lower surface energy of {001} compared with {110} at the higher temperature. {111} faceting is minimal in the low-temperature regime and nearly vanishes when the temperature is increased to 550 °C. In fact, clearly defined {111} facets did not form until the NPs were annealed at 600 °C for 40 min. As the NP surface electronic structure is dependent on the atomic coordination, directly observing the evolution of surface faceting on alloy NPs provides valuable insight for designing NPs with desirable surface configurations.

### Nucleation and development of ordered framework

**Figure 6:**
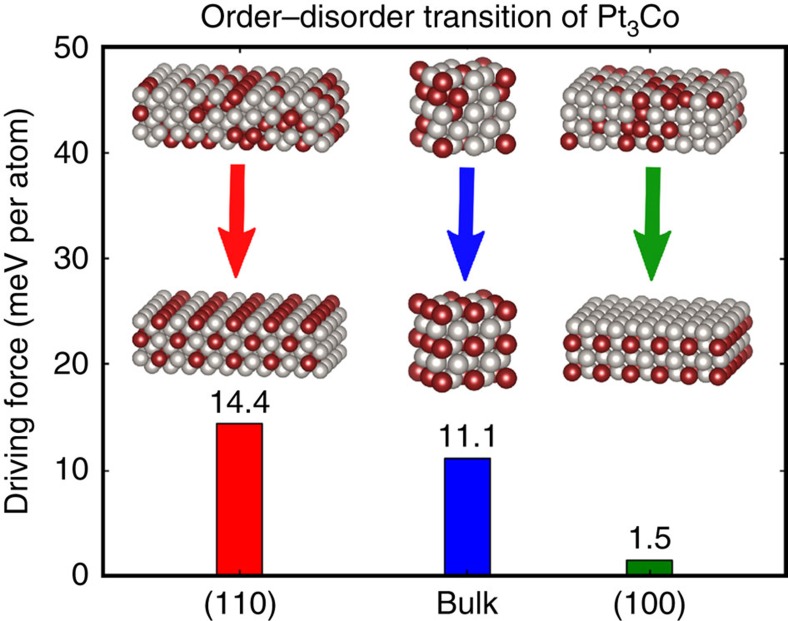
Driving force for elemental ordering. The driving force to form ordered structures at different crystallographic facets and in the bulk determined from using first-principles density functional theory calculations, which show that the (110) facet has the highest driving force to form ordered structure.

When the sample temperature was increased to 600 °C and held for 20 min, alternating bands of bright-dark intensity on the {001} planes appeared at the {110} surface facets of the NP (shown by arrows in Fig. 2d). This alternating contrast is associated with the initiation of elemental ordering, that is, nucleation of the ordered Pt_3_Co phase on {001}. The brighter bands correspond to Pt-rich {001} planes, whereas the darker {001} planes have higher Co concentrations, for example, formation of the ordered superlattice. The observation that Pt-Co ordering initiates at the {110} surfaces *suggests* that this surface has the lowest activation energy for the phase transformation from simple disordered face-centered cubic Pt_3_Co to the ordered primitive L1_2_ Pt_3_Co phase. This is the first direct observation of the surface initiation of the disorder-to-order phase transformation in a single NP, whereby the specific surface associated with the transformation is identified experimentally. However, a critical question is raised: why does the disorder-to-order phase transformation initiate on the Pt_3_Co NP {110} surface facets? First-principles density functional theory (DFT) calculations were performed to evaluate the formation energy between the ordered and disordered phases for a Pt_3_Co bulk crystal, and the dominant surface facets observed for the Pt_3_Co NPs, e.g., {100} and {110}. Energy calculations for these crystallographic surfaces were performed using surface slab models with a thickness of eight atomic layers. The ordered configurations had lower formation energies for all three pairs of comparative structures, 14.4, 11.1 and 1.5 meV per atom for the (110), Pt_3_Co bulk crystal and (100) surfaces, respectively (Fig. 6). Consequently, the energetic driving force for the disorder-to-order transition is the highest for the {110} surfaces of the Pt_3_Co NPs, which matches the experimental observation that atomic ordering of the Pt_3_Co NP initiates on the {110} facets as compared with the {100} facets and the bulk Pt_3_Co NP core.

**Figure 7:**
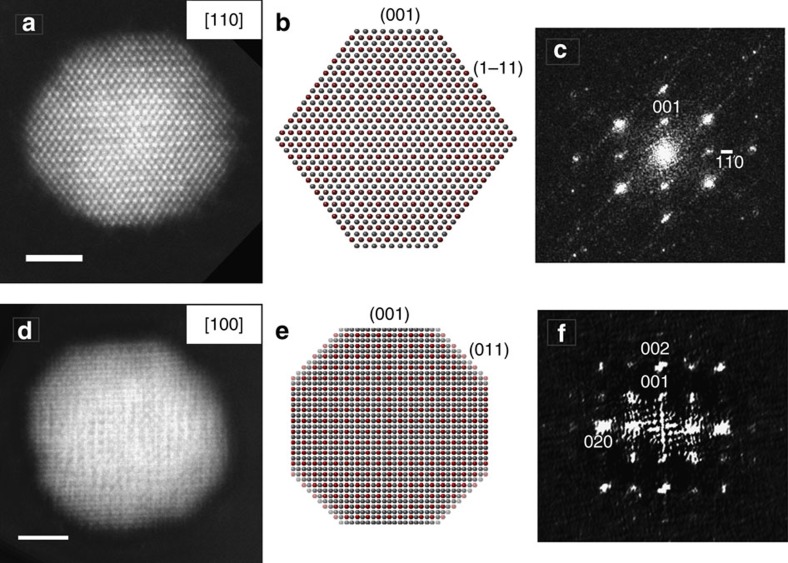
Ordered atomic structures of NPs. (**a**,**d**) HAADF-STEM images of the ordered phase shown for [110] and [100] NP zone axis orientations. (**b**,**e**) Corresponding projections of the perfectly ordered structure models (silver spheres represents Pt atoms and red spheres represent Co atoms). (**c**,**f**) FFTs from the HAADF-STEM images showing superlattice reflections indicative of structural/elemental ordering. Scale bar, 2 nm.

The ordered structures that initiate on the {110} surfaces propagated on the {001} planes into the centre of the NP with further annealing at 600 °C (Fig. 2e). When the particle was annealed at 600 °C for longer times (Fig. 2d–g), elemental diffusion within the bulk progressed, as evidenced by the largely inhomogeneous image contrast intensity distributions (Fig. 2f). The ordered structures form on opposite sides of the NP (Fig. 2d–f), that is, those associated with {110} surfaces, eventually coalescing in the centre of the NP with continued annealing at 600 °C, resulting in an ordered Pt_3_Co NP structure (Fig. 2g). This phase transformation mechanism is consistently observed for multiple Pt_3_Co NPs under these annealing conditions. Figure 7 shows HAADF-STEM images of the annealed Pt_3_Co NPs and corresponding diffractograms (fast Fourier transforms—FFTs—of the images). The <110> oriented NP (Fig. 7a–c) exhibits a different ordering contrast compared with the NP in a <100> zone axis orientation (Fig. 7d–f) as a result of the distinctive elemental stacking in the projected viewing directions. When oriented along <100>, each Co column is surrounded by eight Pt columns, forming a checkerboard-type intensity pattern in the HAADF-STEM image (Fig. 7d,e). The {100} superlattice reflections are clearly observed in the corresponding FFTs (Fig. 7f). It should be noted that the disorder-to-order phase transformation temperature of 600 °C for the Pt_3_Co NPs is slightly lower than that reported for bulk Pt_3_Co (∼650 °C)^18^, which can be explained by a higher elemental inter-diffusion rate within NPs compared with that for the bulk material owing to the significantly increased surface area of the NPs^19^.

### Initiation of amorphization

Continued annealing at 600 °C for another 20 min does not change the ordering architecture but leads to further elemental inter-diffusion (Fig. 2g). Complete ordering within the Pt_3_Co particle is realized when the temperature is increased to 700 °C and held for only 10 min. (Fig. 2h). The contrast difference between the ordered {001} planes (alternating Pt-rich and Co-rich layers) is obviously greater compared with that observed at 600 °C, indicating extensive Pt-Co inter-diffusion towards the optimized L1_2_ ordered structure. No observable elemental diffusion or structure changes were observed with further annealing at 700 °C for 30 min, which suggests that the NP achieved a thermally stable L1_2_ structure after 10 min. Our observations on the evolution of the ordered structure shows that an ordered framework is established first in the NPs followed by additional diffusion towards a stable ordered structure. When the Pt_3_Co NPs are further annealed to a temperature of 800 °C, the atomic columns in the HAADF-STEM image (Fig. 2i) become blurred as a result of increased atomic vibration during imaging at the elevated temperature, although the ordered structure is still vaguely visible. Longer annealing times at 800 °C results in amorphization (that is, pre-melting) of the NP and the morphology assumes a rounded shape and loss of surface faceting.

The progressive transition of the disordered alloy Pt_3_Co NP to the ordered L1_2_ phase (200–700 °C) and subsequent loss of crystallinity (800 °C) is also shown by the evolution of the diffractograms (FFTs). Supplementary Fig. 5 shows a series of FFTs acquired from selected HAADF-STEM images shown in Fig. 2. The intensity of the superlattice reflections ({001} and {110}) can be used as a direct indicator of the extent of structural ordering in the Pt_3_Co NP. The superlattice reflections appeared initially for the NP annealed at 600 °C for 20 min (Supplementary Fig. 5c). Continued annealing results in the gradual increase in the intensity of the superlattice reflections (Supplementary Fig. 5c,d). When the NP is annealed at 700 °C for 10 min (Supplementary Fig. 5e), the superlattice reflections are sharp and intense, corresponding to a highly ordered L1_2_ structure. Annealing to 800 °C results in diffuse diffraction spots (Supplementary Fig. 5f), which may result from the vibration of atomic columns before amorphization of the structure, although the NP might be slightly tilted away from [110] zone axis indicated by the loss of {011} superlattice reflections in the FFT (Supplementary Fig. 5f).

## Discussion

The detailed three-stage progression of a random fcc alloy Pt_3_Co NP to the L1_2_ phase during annealing is characterized by ordered domain nucleation on {001} preferentially at the {110} surface facets, establishment of the ordered framework through the gradual ordering of Pt and Co on {001} planes, and optimization of the L1_2_ phase through continued elemental inter-diffusion. Understanding and controlling this phase transition can aid the design of NPs with exquisite control of the atomic structure and extent of ordered architectures to attain specific electronic structures. Furthermore, the response of elemental inter-diffusion within the NPs to the annealing temperature and time demonstrates that the phase transformation is both a kinetic and dynamic process; for example, a particular temperature is required to thermally activate elemental diffusion, whereas the annealing time is important for allowing the atomic movements to be completed.

**Figure 8:**
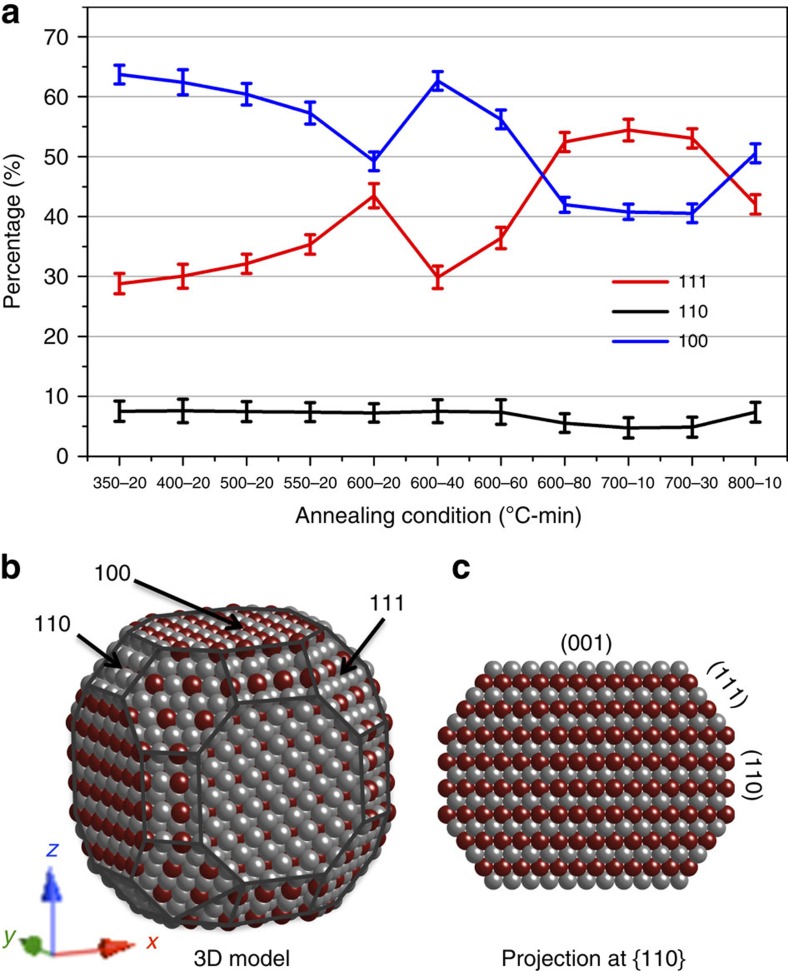
Evolution of NP surface facets. (**a**) The evolution of the concentrations of the surface facets, for example, {111}, {110} and {100}, as a function of annealing condition; the concentrations were quantified by assuming a truncated cuboctahedron NP morphology; error bars represent the s.d. resulting from five measurements of the facet sides. (**b**,**c**) Detailed geometrical correlation between the facets and the two-dimensional projection down [110].

The interplay between elemental diffusion, surface faceting and phase transformation ultimately controls the catalytic performance of NPs^20^,^21^. The dynamic structural and elemental changes during thermal annealing can be directly visualized through high-resolution HAADF-STEM imaging (Fig. 2). The {110} planes were found to be the dominant surface facets formed in the early stages of annealing (550 °C) and were also the surface facets associated with the initiation of structural ordering. However, the area of the {110} surfaces gradually diminished with further elemental inter-diffusion (600 °C, Fig. 2d–g)), and eventually reached the minimum size of 2–3 unit cells in width at 700 °C (Fig. 2h), while simultaneously, structural ordering continued until a fully ordered L1_2_ structure was achieved (Fig. 2h). Concurrently, the {111} facets became the dominant surface (Fig. 2g,h) at the expense of {110} facets at the higher temperatures, indicative of a higher surface energy in the ordered L1_2_ phase compared with the {111} and {100} facets during annealing, even though {110} was the preferred surface for the nucleation of the ordered domains. When the NPs were further annealed to 800 °C, {110} facets reformed and the overall NP morphology evolved to a truncated cuboctahedron. The evolution of the surface area of each facet type enclosing the NP, that is, {111}, {110} and {100}, was assessed as a function of annealing conditions, as summarized in Fig. 8. This quantification is based on an assumption that the NPs hold a truncated cuboctahedron shape during annealing, a shape composed of 8 {111}, 12 {110} and 6 {100} facets, as shown in Fig. 8b,c (see details in Supplementary Information). The results shown in Fig. 8a clearly demonstrate the dynamic nature of NP morphology development throughout the annealing process, resulting from the competition between the surface energies of the facets. It is interesting to note that the NP exhibited the largest {111} surface area when the L1_2_ phase was completely formed (700 °C for 10 min), such that the highest catalytic activity is expected if the facets in the L1_2_ phase follow the same rule as the completely disordered alloy (random solid solution phase)^22^. These results demonstrate that the preferred NP facets for improved catalytic activity can be designed through a careful selection of annealing conditions. Moreover, as surface faceting behaviour is strongly coupled with elemental inter-diffusion, the interplay between these two phenomena should be considered during materials design. Such NP morphological optimization is established by combining surface elemental distribution and ordering (Fig. 2) with the specific type of facet and area evolution (Fig. 8).

It should be noted that the surface faceting and diffusion behaviours upon annealing may vary under different gas environments depending on how much the thermodynamic driving force in the Pt-Co binary system is altered. For example, annealing Pt_3_Co NPs under forming gas at ambient pressure most likely does not alter the faceting and elemental diffusion behaviour, because neither Pt nor Co form metal hydrides when annealed in H_2_, and the adsorption of hydrogen on Pt or Co is rather weak^23^,^24^. In contrast, an oxidizing atmosphere might significantly influence the elemental diffusion behaviour in alloyed NPs^25^. *In situ* atomic-scale investigations under different gas environments of bi-metallic catalysts will be interesting and important; in future work, we will study these phenomena using a closed-cell gas-reaction system (Protochips Atmosphere 200).

In summary, our study directly reveals the dynamic structural and chemical evolution at the atomic scale of Pt_3_Co NPs during thermal annealing. The nanocatalysts exhibit distinct elemental diffusion behaviour at critical annealing stages: the formation of a Pt-rich shell with a randomly alloyed core; the preferential nucleation of ordered domains associated with specific surface facets; the establishment of a fully ordered L1_2_ phase and, finally, pre-melting. The dynamic coupling of surface faceting and elemental inter-diffusion during annealing was also discovered, which should be carefully considered during catalyst design. The major facets, {111}, {110} and {100}, play differing roles during evolution of the NP structure and chemistry. Our results provide insight regarding the rational design of NPs at the atomic scale aimed at creating enhanced catalytic activity and stability through optimization of the surface structure and elemental distribution using thermal annealing. Finally, our studies demonstrate the power of directly tracking the atomic configurations, including both atomic position and chemical distribution, within single particles during *in situ* annealing using sub-Ångström-resolution and chemically sensitive HAADF-STEM imaging.

## Methods

### Specimen preparation and microscopy experiments

The Pt_3_Co NPs were synthesized through an organic solvothermal approach, including dissolving Pt(acac)_2_ in oleylamine and benzyl ether, heating the formed solution, injecting dissolved Co_2_(CO)_8_, cooling and centrifuging. The detailed synthesis was described in our earlier papers^11^. The Pt_3_Co NPs derived from organic solvothermal synthesis were loaded on an amorphous carbon support. The STEM specimens were prepared by dispersing the supported NPs in a hexane solution and then onto Protochips heating devices. To minimize possible surface contamination on NPs, the STEM specimen was pumped in vacuum for ∼10 h before STEM experiments.

STEM imaging experiments were carried out on an aberration-corrected JEOL 2200FS microscope fitted with a CEOS GmbH aberration corrector on the probe-forming lenses, and a BrukerAXS X-Flash Si drift detector for EDS. Protochips Aduro heating technology^26^ was used for the *in situ* heating experiments to control the temperature. The specimen was heated from 200 to 800 °C using 50 °C s^−1^ increments, and 30 min holding time at each temperature. The annealing time at 600 °C was 100 min in order to have time for a detailed observation of elemental segregation behaviour. Twenty-five NPs in total were tracked upon the annealing. Such *in situ* experiments were implemented twice to confirm the evolution of atomic structure in the NPs. Energy-dispersive X-ray elemental maps were acquired from several NPs with a beam current of 40 pA, from RT to 550 °C at elevated temperatures by using the Bruker Si drift detector system. EDS mapping above 550 °C was not achievable because of hardware limitations. All EDS maps were acquired from a region away from that for imaging in order to minimize electron beam effects on surface atomic structure and chemistry. The specimen was exposed to the electron beam only during data acquisition.

The HAADF images were recorded at 200 kV using a convergence angle of 26.5 mrad and an inner and outer collection angles of 110 and 470 mrad, respectively. To minimize beam irradiation, a relatively small beam current of 20 pA was used for imaging.

### Image contrast and its chemical sensitivity

Because the contrast of the HAADF images is proportional to average atomic number (that is, ∼Z^1.7^), the intensity of each atomic column is a combined result from both elemental atomic number and local thickness, that is, the number of atoms at a particular viewing projection. Assuming a truncated cubo-octahedron shape of the NPs, the elemental concentrations of Pt and Co at each atomic column can be estimated directly using the image contrast, owing to the large difference in their atomic numbers^15^,^16^. As a result, the contrast of atomic columns can be used to monitor elemental diffusion in this work, such as Figs 3 and 4 and Supplementary Fig. 4.

### Construction of structural models of Pt_3_Co NPs

A Pt_3_Co NP with a diameter of 5.2 nm and with randomly distributed Pt and Co atoms in a cubo-octahedron geometry was first constructed. The overall Pt concentration of the particle was about 75 at.% (Supplementary Fig. 6a). Based on this initial particle structure, structural relaxation was applied to resolve the relaxed atomic model (Supplementary Fig. 6b). Furthermore, energy-biased Metropolis MC simulations at a temperature of 623 K (350 °C) were used to obtain a Pt-segregated NP (Supplementary Fig. 6c). In the MC simulation, we attempted to transform the configuration of the NP through exchanging the positions of one Pt and another Co atom, as well as through displacing the positions of the Pt and Co atoms until the simulated NP reached its thermodynamically equilibrated states.

### HAADF image simulation method

After the atomic models of Pt_3_Co NPs with and without Pt segregations (Supplementary Fig. 6c,b) were established using simulations, their atomic coordinations were fed to the crystal building software CrystalMaker, to generate a representative atomic model. A quarter of the atoms in the [110] zone axis direction, resulting in a supercell of 2.5 × 2.5 nm^2^, were extracted from each model for image simulations, as shown in Fig. 4. The HREM Simulation Suite^27^, which is based on the FFT Multislice technique, was used for HAADF-STEM simulation. Such simulation software evaluates both the wave function and its Fourier transform at each slice, using the approximation by Weickenmeier and Kohl to calculate the elastic scattering amplitude. The simulation was carried out using a 128 × 128 pixel area and a slice thickness of 1.0 Å. Thermal diffuse scattering factors, 0.008 Å^2^ for Co and 0.007 Å^2^ for Pt, were considered into the elastic scattering amplitude to obtain atomic intensity in HAADF images. The microscopy parameters used for the simulations were the same as those for imaging, and are shown in Supplementary Table 1.

### Density functional theory calculations

In this study, the energies of ordered and disordered Pt_3_Co alloys were calculated using software VASP^28^,^29^. In all the DFT calculations, Perdew–Burke–Ernzerhof functional^30^, projector augmented wave method^31^ and energy cutoff of 600 eV were used. The ordered Pt_3_Co bulk crystal was modelled using a super cell consisting of 2 × 2 × 2 L1_2_ unit cells and the stoichiometric, ordered Pt_3_Co (001) and (110) surface slabs were modelled using 8 atomic layers (4 atoms in each layer) with alternating chemical compositions of pure Pt and 50% of Pt (50% of Co). The corresponding disordered Pt_3_Co bulk crystal and surface slabs were constructed using the Alloy Theoretic Automated Toolkit^32^. In particular for the two disordered surfaces, multiple surface terminations were identified. Thus, we took the average energy of all the possible surface terminations as the system energy of disordered Pt_3_Co surfaces. In our DFT study, all the structures were relaxed until the Hellman–Feynman force exerted on each atom was less than 0.01 eV Å^−1^.

### Evaluation of facet evolution

The evolution of facets, that is, {100}, {110} and {111}, was assessed based on an assumption that the NP holds a truncated cubo-octahedron shape. Such morphology is also evidenced in STEM images (Fig. 2). A truncated cubo-octahedron contains 8 × {111}, 6 × {100} and 12 × {110} facets. The outlines of the [110] projection comprise the information of the facet dimensions. A three-dimensional model of truncated cubo-octehedron particle and its corresponding two-dimensional projection are shown in Supplementary Fig. 7. During annealing, facets evolve and the particle no longer preserves an icosohedron shape. We thus considered this factor in our evaluations by treating each facet area with a reduced symmetry. For example, instead of assuming {110} facets in a square area, a rectangular shape was considered. All edges revealed in [100] projection were measured and considered in the evaluation. The detailed estimations are shown in supplementary Fig. 7.

## Additional information

**How to cite this article:** Chi, M. *et al*. Surface faceting and elemental diffusion behaviour at atomic scale for alloy nanoparticles during *in situ* annealing. *Nat. Commun.* 6:8925 doi: 10.1038/ncomms9925 (2015).

## Supplementary Material

Supplementary Information Supplementary Figures 1-7 and Supplementary Table 1
