# Supplementary material for: Surface faceting and elemental diffusion behaviour at atomic scale for alloy nanoparticles during in situ annealing
Source: Nat Commun. 2015 Nov 18;6:8925. doi: 10.1038/ncomms9925 (PMC4673855; doi:10.1038/ncomms9925)
Supplement: Supplementary Information — Supplementary Figures 1-7 and Supplementary Table 1 [file ncomms9925-s1.pdf]

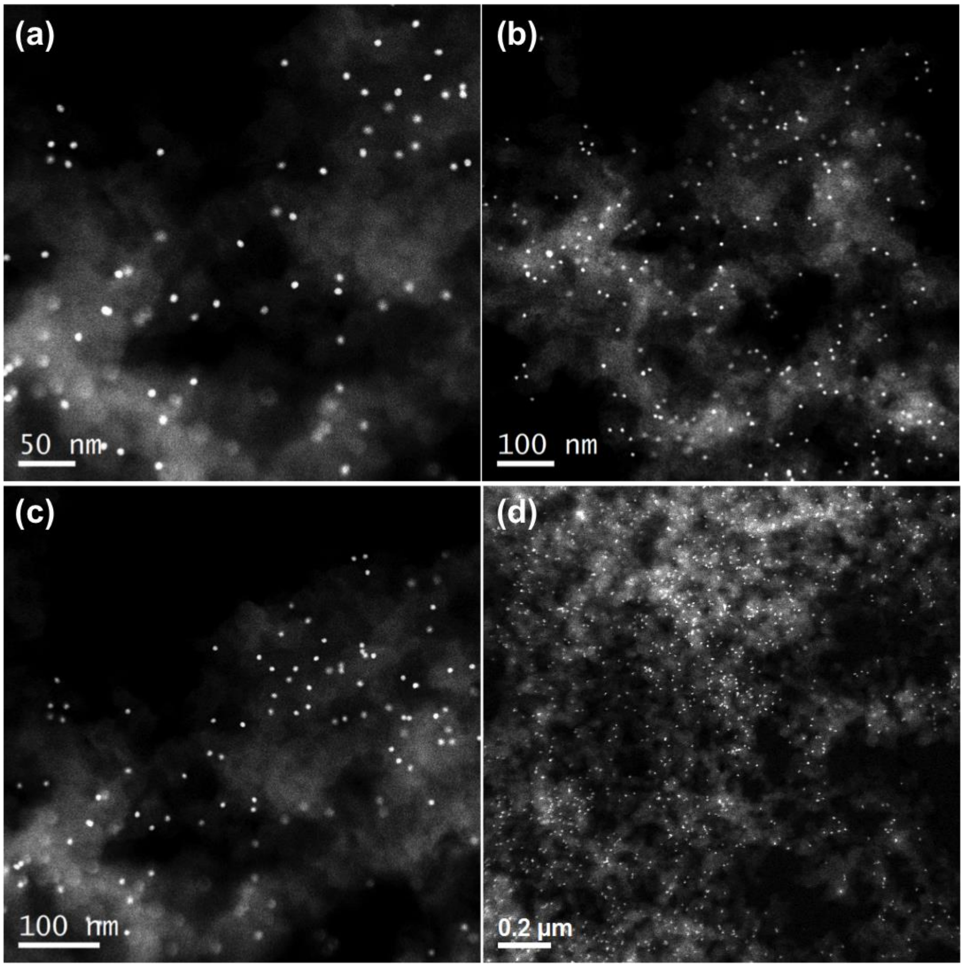

Supplementary Figure 1. (a)-(d) HAADF images of pristine Pt_3Co NPs show excellent size homogeneity and monodispersivity on supporting carbon.

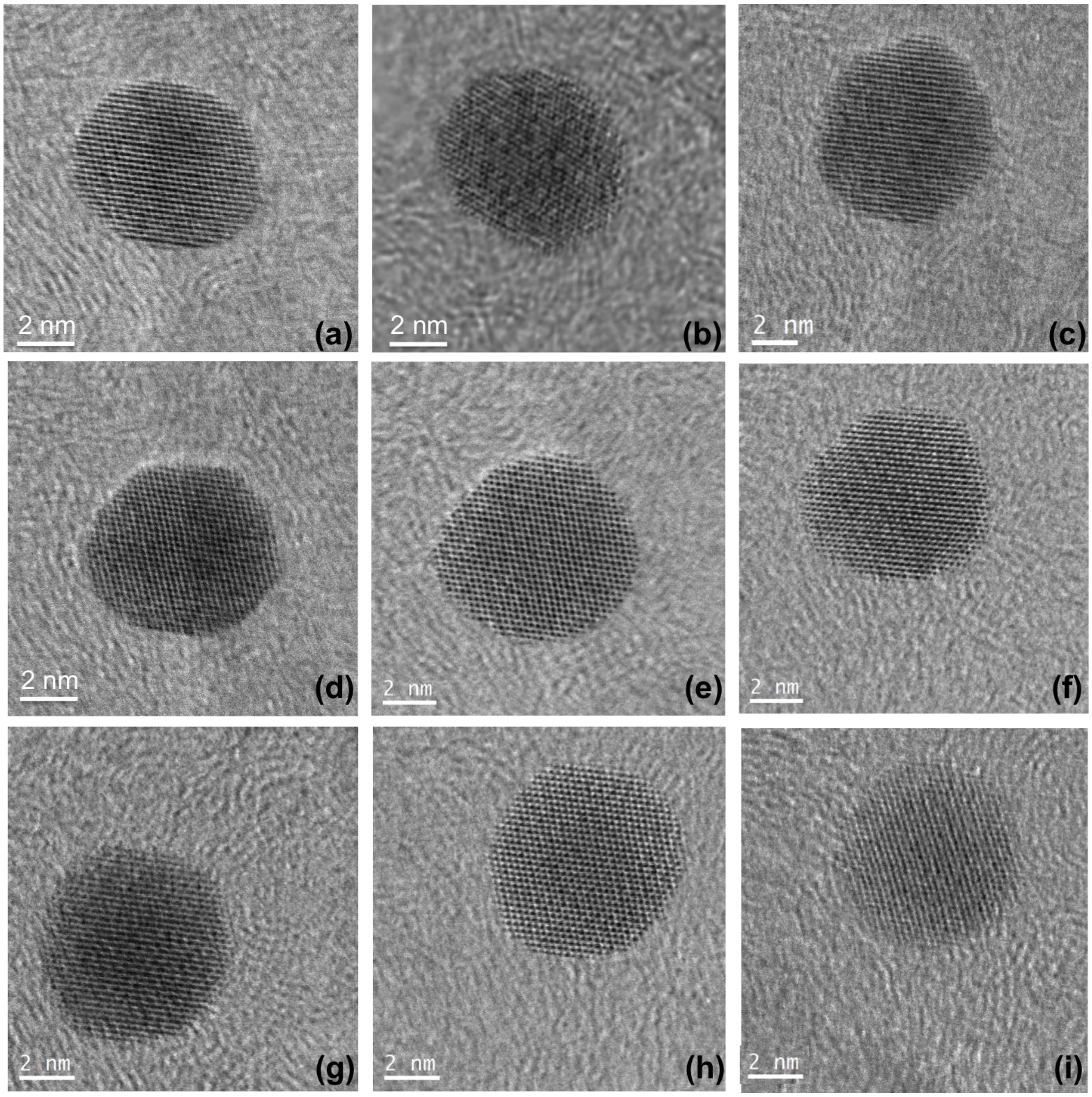

Supplementary Figure 2. Atomic-resolution bright-field (BF) STEM images of the single Pt_3Co NP that were acquired simultaneously with the HAADF-STEM images in Fig. 2.

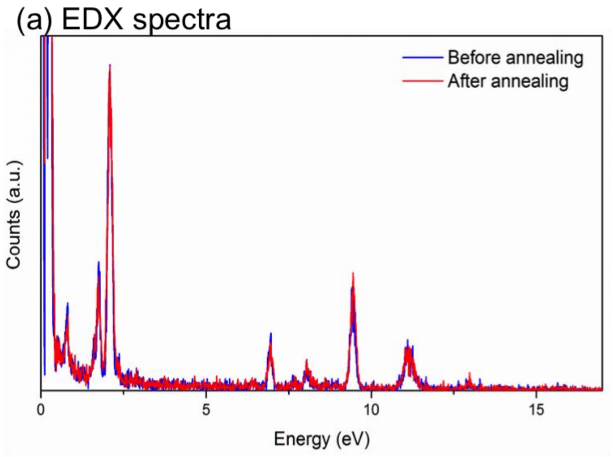

(b) EDX quantifications of spectra in (a)

Element	series	[norm. wt.%]	[norm. at. %]	Error in at. %
**Before annealing**				
Cobalt	K-series	10.87	28.76	1.78
Platinum	L-series	89.13	71.24	7.51
	Sum:	100	100	0.00
**After annealing**				
Element	series	[norm. wt.%]	[norm. at. %]	Error in at. %
Cobalt	K-series	8.73	24.05	1.49
Platinum	L-series	91.27	75.95	7.91
	Sum:	100	100	

Supplementary Figure 3. Energy-dispersive X-ray spectra of the NP in Fig. 2 pre- and post-annealing. Both spectra were acquired at RT; the post-annealing spectrum was acquired after the particle was quenched from 800°C to RT. Both the spectra (a) and the corresponding elemental quantification (b) show no detectable chemical loss on the NP.

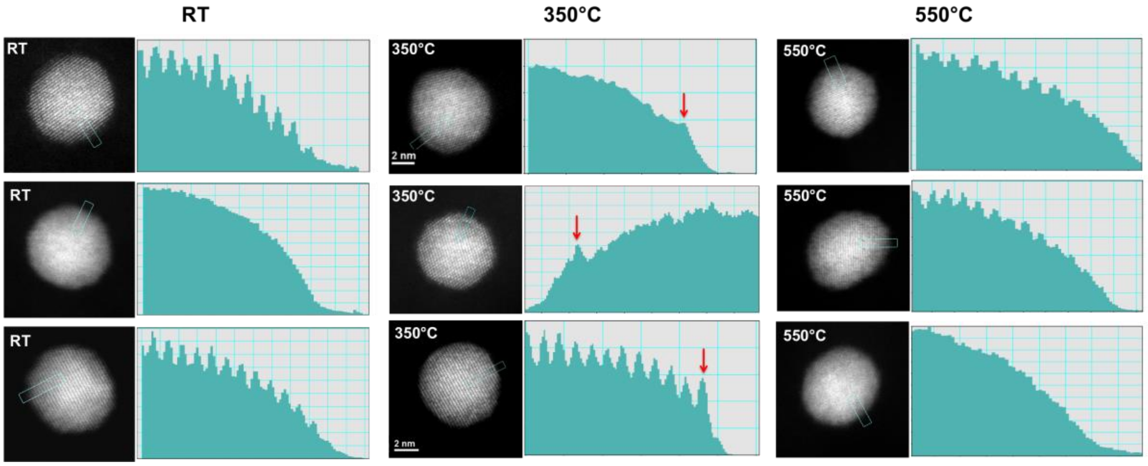

Supplementary Figure 4. Additional representative Pt_3Co NPs showed the same segregation behavior at different annealing temperatures as exhibited by the particle in Figs. 2 and 3. The enhanced contrast at the surface region was observed on the 350°C annealed particles, as marked by arrows on the profiles.

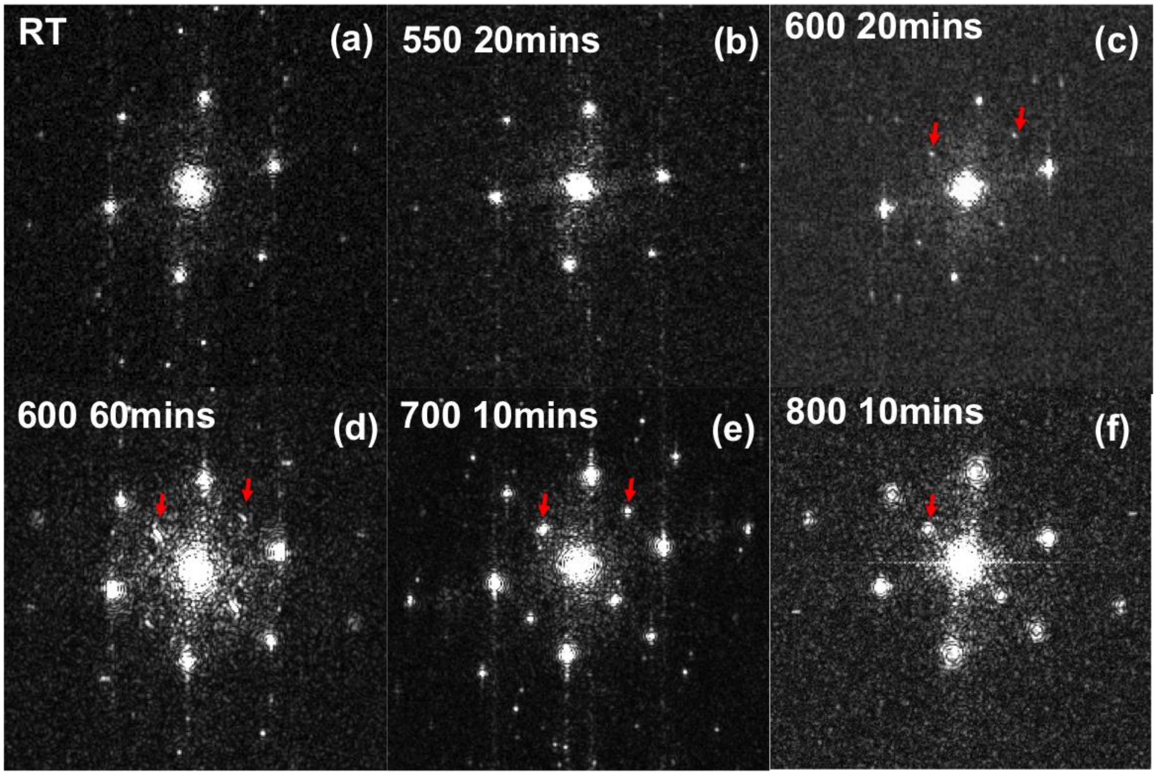

Supplementary Figure 5. Fast Fourier transforms computed from selected high-resolution images in Fig. 2. The representative ordering periodicities ½{001} and ½ {011} are indicated by arrows.

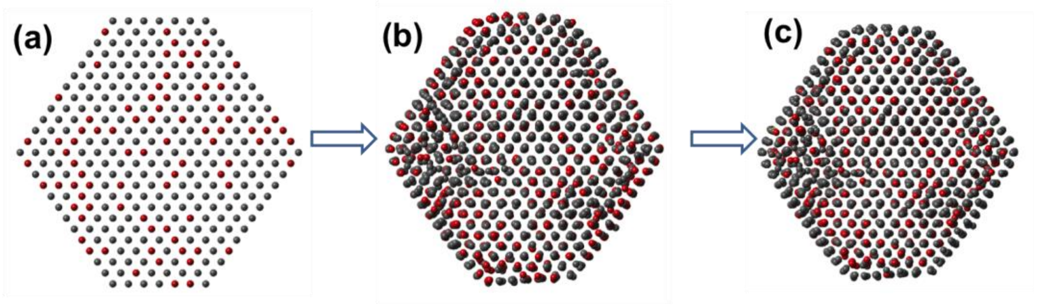

Supplementary Figure 6. calculated structural models of Pt_3Co NPs for multi-slice STEM image simulations: randomly alloyed NP in a cubo-octahedron shape (a); after structural relaxation (b); and with Pt segregation (c).

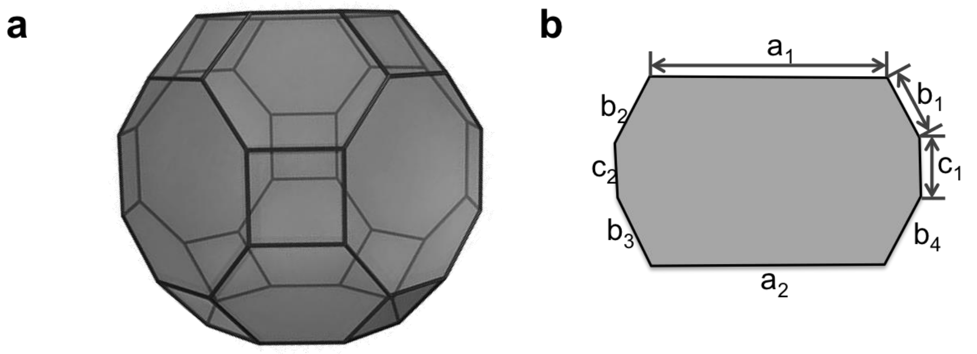

3-D model of truncated cubooctahedron

2-D projection at [110], and the edges for the calculation were labeled.

c

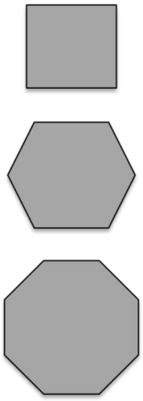

12X 110

$$S_{(110)} = 4(xy + yz + xz) \qquad \text{............ (1)}$$

8X 111

$$S_{(111)} = 8 \times 3 \times \frac{\sqrt{3}}{2} y^2 \qquad \text{............ (2)}$$

6X 100

$$S_{(100)} = 3[(1 + 2\sqrt{2})(a^2 + c^2) + ab + ac] \qquad \text{............ (3)}$$

$$x = \frac{a}{(1 + \sqrt{2})} \qquad \text{............ (4)}$$

$$y = b \qquad \text{............ (5)}$$

$$z = \frac{c}{(1 + \sqrt{2})} \qquad \text{............ (6)}$$

Supplementary Figure 7. Schematic of facets and calculation equations that are used for estimating enclosing facet area of the NP. The 3D schematic model of truncated cubooctahedron (a) and its 2-D projection at [110] direction (b); the lengths of a_i, b_i, and c_i (i=1,2,3…) are measured from STEM images for facet evaluations. A truncated cubo-octahedron contains 8X{111}, 6X{100} and 12X{110} facets (c). x, y, and z are

the side lengths of the (100) facets, and a, b, and c are the averages from the measured a_i, b_i, and c_i values respectively. The area of surface facets are calculated using equation (1)-(3), and the mathematic relationship between x, y, z and a, b, c are shown in equation (4)-(6).

Supplementary Table 1. Microscopy parameters for image simulation

**Voltage (kV)**	**Convergent angle**	**C1**	**A1**	**A2**	**B2**	**C3**	**A3**	**A4**
200	30 mrad	700 pm	2.22 nm	35.5 nm	30.4 nm	100.1 nm	264.7 nm	8.1 um
